# Supplementary material for: Structural basis for pH-responsive amino acid transport via SLC7A4
Source: Nat Commun. 2026 Mar 28;17:4544. doi: 10.1038/s41467-026-70956-5 (PMC13194723; doi:10.1038/s41467-026-70956-5)
Supplement: Supplementary file 1 — Supplementary Information [file 41467_2026_70956_MOESM1_ESM.pdf]

**Structural basis for pH-regulated amino acid transport via SLC7A4.**

Dimitrios Kolokouris[1,2,3], Anuja Bothra[1,2], Takafumi Kato[1,2], Yi C. Zeng[1,2], Simon Lichtinger[1,3], Joanne L. Parker[1,2], Philip Biggin[1,3], Simon Newstead[1,2*].

[1] Department of Biochemistry, University of Oxford, Oxford, OX1 3QU, UK.
[2] Kavli Institute for Nanoscience Discovery, University of Oxford, Oxford, UK.
[3] Structural Bioinformatics and Computational Biochemistry, Department of Biochemistry, University of Oxford, Oxford, UK.

* simon.newstead@bioch.ox.ac.uk

Supplementary Fig. 1-12
Supplementary Table 1: Cryo-EM Data Collection, Processing and Refinement Statistics.
Supplementary Table 2: Molecular Dynamics Data Table.

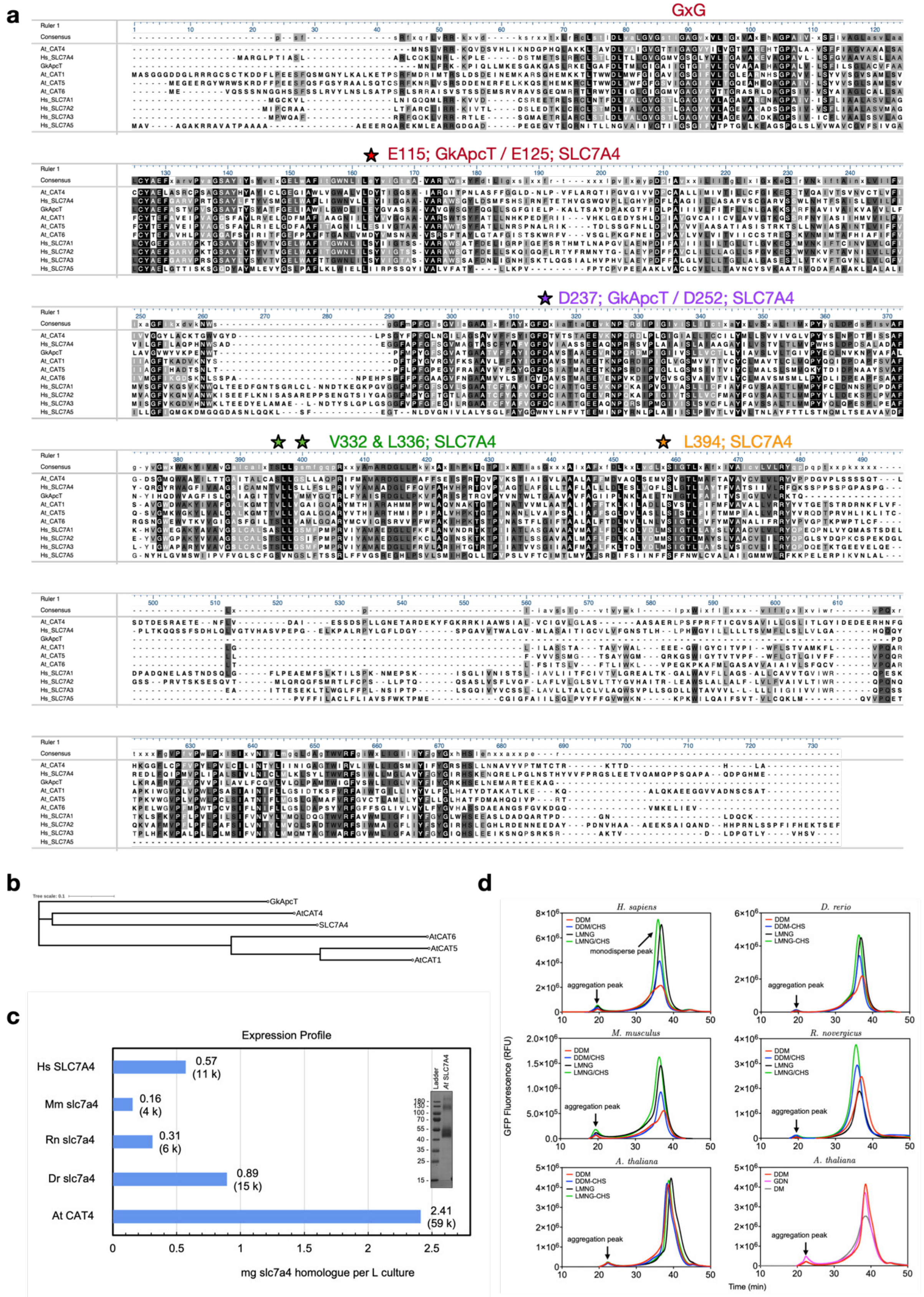


**Supplementary Fig. 1. Sequence analysis and homologue screening for SLC7A4. a.** Sequence alignment of SLC7A4/CAT homologues (At_CAT4 Uniprot: Q8W4K3; Hs_SLC7A4: O43246; GkApcT: Q5L1G5; At_CAT1: Q84MA5; At_CAT5:

O64759; At_CAT6: Q9LZ20; Hs_SLC7A1: P30825; Hs_SLC7A2: P52569; Hs_SLC7A3: Q8WY07; Hs_SLC7A5: Q01650). **b.** Multiple sequence alignment generated with T-COFFEE for a phylogenetic tree. Pairwise distances were calculated in EMBL-EBI Simple Phylogeny using neighbour-joining with distance correction enabled, gaps included. The resulting tree (PHYLIP format) was visualised and annotated using iTOL (Interactive Tree Of Life). **c.** Expression data for recombinant production of SLC7A4 homologues in *S. cerevisiae*. Inset – SDS PAGE gel of purified AtCAT4 used for the structural and biophysical studies. **d.** Fluorescent SEC profiles for the SLC7A4 homologues are displayed in panel a.

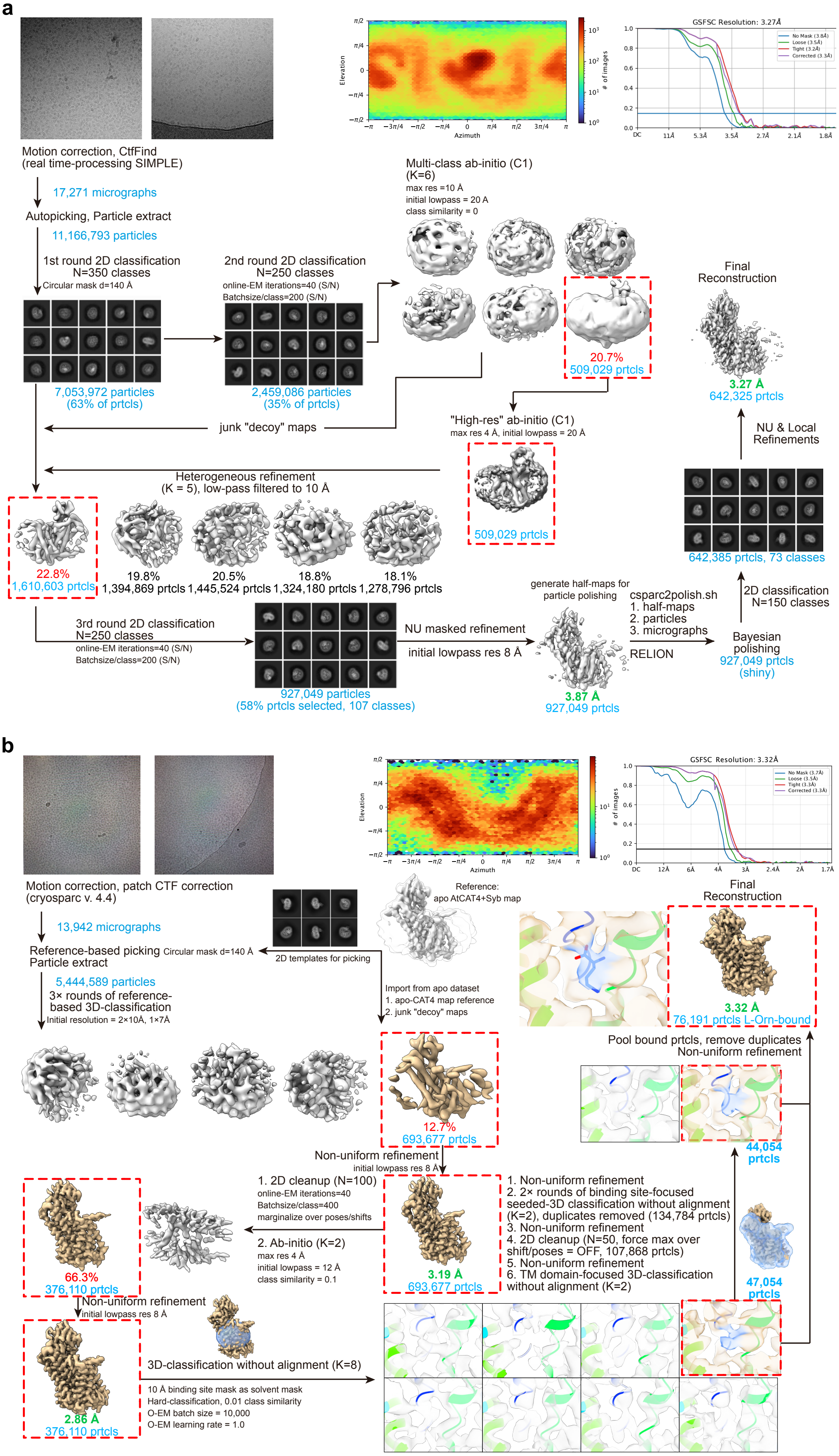
a
Motion correction, CtfFind
(real time-processing SIMPLE)
17,271 micrographs
Autopicking, Particle extract
11,166,793 particles
1st round 2D classification
N=350 classes
Circular mask d=140 Å
7,053,972 particles
(63% of prtcls)
2nd round 2D classification
N=250 classes
online-EM iterations=40 (S/N)
Batchsize/class=200 (S/N)
2,459,086 particles
(35% of prtcls)
Multi-class ab-initio (C1)
(K=6)
max res =10 Å
initial lowpass = 20 Å
class similarity = 0
20.7%
509,029 prtcls
junk "decoy" maps
"High-res" ab-initio (C1)
max res 4 Å, initial lowpass = 20 Å
509,029 prtcls
Heterogeneous refinement
(K = 5), low-pass filtered to 10 Å
22.8%
1,610,603 prtcls
19.8%
1,394,869 prtcls
20.5%
1,445,524 prtcls
18.8%
1,324,180 prtcls
18.1%
1,278,796 prtcls
3rd round 2D classification
N=250 classes
online-EM iterations=40 (S/N)
Batchsize/class=200 (S/N)
927,049 particles
(58% prtcls selected, 107 classes)
NU masked refinement
initial lowpass res 8 Å
generate half-maps for particle polishing
3.87 Å
927,049 prtcls
csparc2polish.sh
1. half-maps
2. particles
3. micrographs
RELION
Bayesian polishing
927,049 prtcls
(shiny)
2D classification
N=150 classes
642,385 prtcls, 73 classes
NU & Local Refinements
Final Reconstruction
3.27 Å
642,325 prtcls
GSFSC Resolution: 3.27Å
No Mask (3.8Å)
Loose (3.5Å)
Tight (3.2Å)
Corrected (3.3Å)
Elevation
Azimuth
# of images
b
Motion correction, patch CTF correction
(cryosparc v. 4.4)
13,942 micrographs
Reference-based picking Circular mask d=140 Å
Particle extract
2D templates for picking
Reference:
apo AtCAT4+Syb map
5,444,589 particles
3× rounds of reference-based 3D-classification
Initial resolution = 2×10Å, 1×7Å
Import from apo dataset
1. apo-CAT4 map reference
2. junk "decoy" maps
12.7%
693,677 prtcls
Non-uniform refinement
initial lowpass res 8 Å
3.19 Å
693,677 prtcls
1. 2D cleanup (N=100)
online-EM iterations=40
Batchsize/class=400
marginalize over poses/shifts
2. Ab-initio (K=2)
max res 4 Å
initial lowpass = 12 Å
class similarity = 0.1
66.3%
376,110 prtcls
Non-uniform refinement
initial lowpass res 8 Å
2.86 Å
376,110 prtcls
3D-classification without alignment (K=8)
10 Å binding site mask as solvent mask
Hard-classification, 0.01 class similarity
O-EM batch size = 10,000
O-EM learning rate = 1.0
1. Non-uniform refinement
2. 2× rounds of binding site-focused seeded-3D classification without alignment (K=2), duplicates removed (134,784 prtcls)
3. Non-uniform refinement
4. 2D cleanup (N=50, force max over shift/poses = OFF, 107,868 prtcls)
5. Non-uniform refinement
6. TM domain-focused 3D-classification without alignment (K=2)
47,054 prtcls
44,054 prtcls
Pool bound prtcls, remove duplicates
Non-uniform refinement
Final Reconstruction
3.32 Å
76,191 prtcls L-Orn-bound
GSFSC Resolution: 3.32Å
No Mask (3.7Å)
Loose (3.5Å)
Tight (3.3Å)
Corrected (3.3Å)
Elevation
Azimuth
# of images

***Figure continued below:***

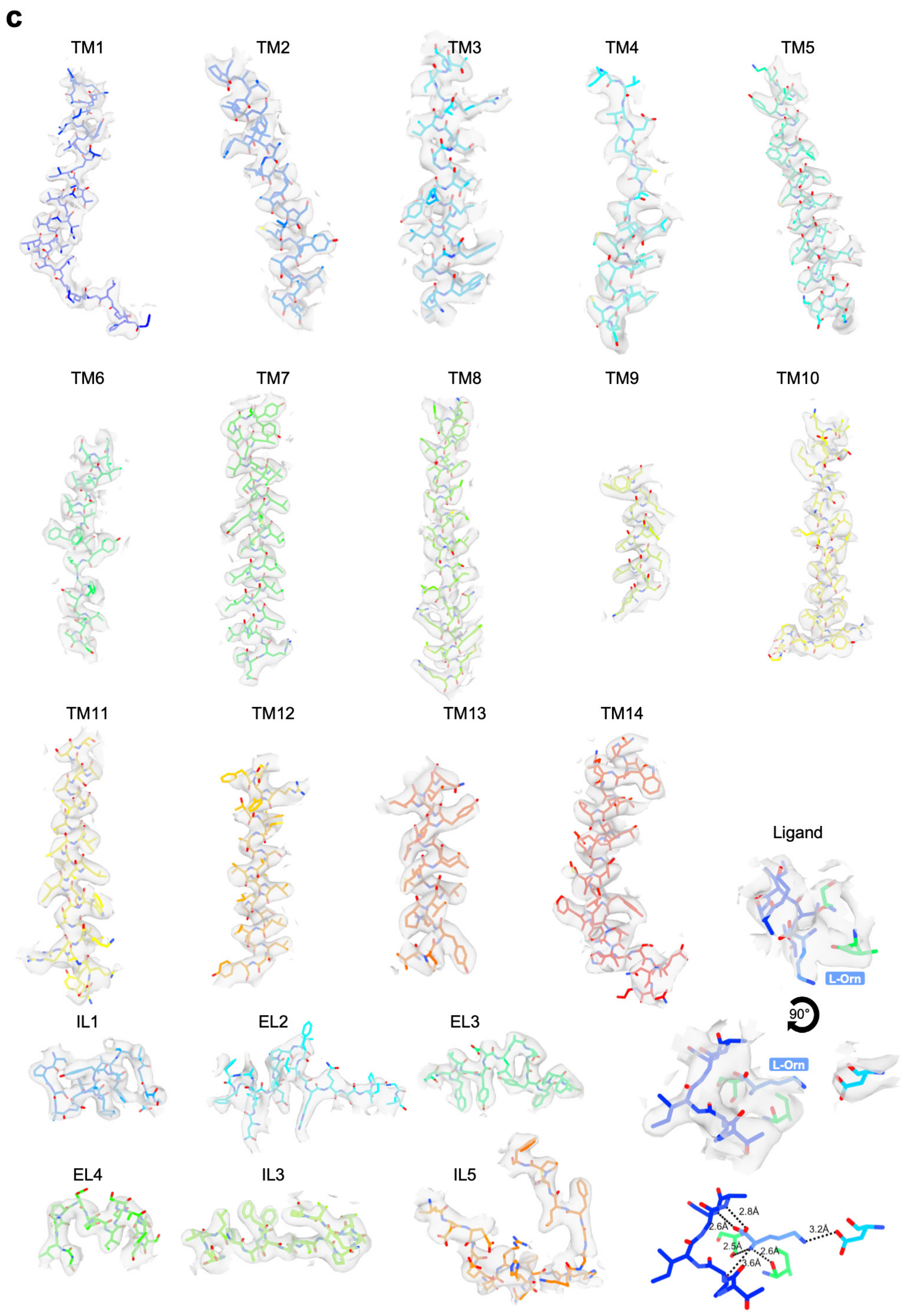


**Supplementary Fig. 2. Cryo-EM processing pipeline for AtCAT4 bound to sybody B5 (SybB5). a.** Cryo-EM processing pipeline for Apo AtCAT4. **b.** Cryo-EM processing pipeline for L-Orn bound AtCAT4. **c.** Atomistic model fitting of Orn+SybB5 bound AtCAT4 dataset (EMDB: 55065). Loops named according to canonical LeuT nomenclature reported, σ level = 6.5

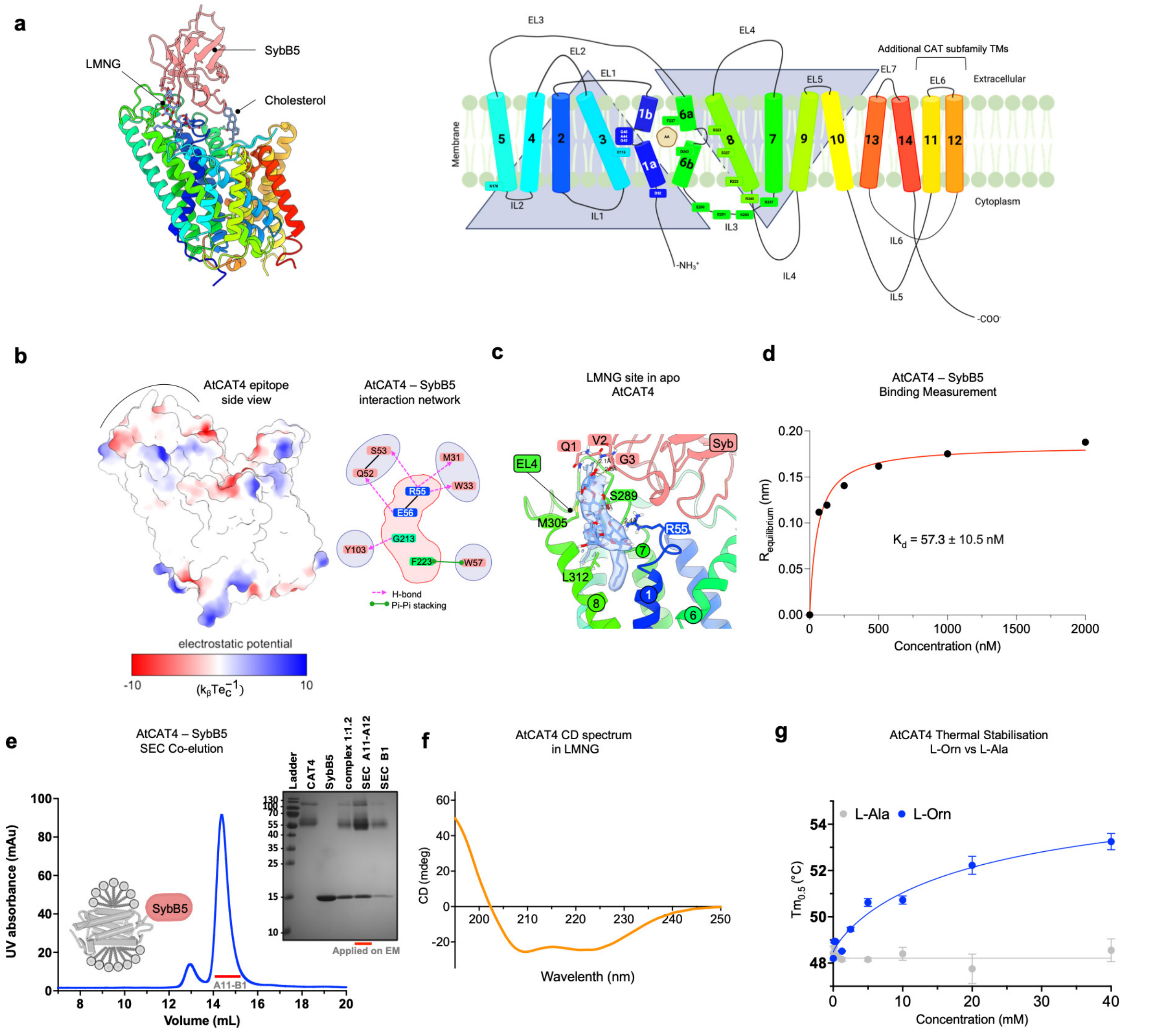

**Supplementary Fig. 3. Cryo-EM structure of AtCAT4 bound to sybody B5 (SybB5). a.** Cryo-EM structure of apo AtCAT4 bound to SybB5 coloured from the N-terminus (blue) to the C-terminus (red). The topology diagram for the structure is shown to the right. The inverted topology repeats (TMs 1-5 & 6-10) are shown as inverted triangles. **b.** Electrostatic surface representation of AtCAT4 showing the structural epitope recognised by SybB5. **c.** LMNG binding interface on apo AtCAT4 bound to SybB5. The cryo-EM density for the bound LMNG molecule is shown (blue and threshold 0.3). **d.** Binding affinity ($K_d$) measurement for SybB5, n=2 independent measurements. **e.** Size Exclusion Chromatography (SEC) profile for the AtCAT4-SybB5 complex using a Superdex S200 10/300 GL Increase column. Inset, SDS-PAGE analysis of the fractions highlighted in the chromatogram. **f.** Circular Dichroism (CD) spectra for the LMNG purified sample used in the cryo-EM experiments. **g.** Thermal stabilisation of the LMNG purified AtCAT4 in the presence of increasing concentrations of L-Ornithine. Increasing concentrations of L-Alanine were used as a negative control. n = three independent experiments; errors shown are standard deviations (s.d.).

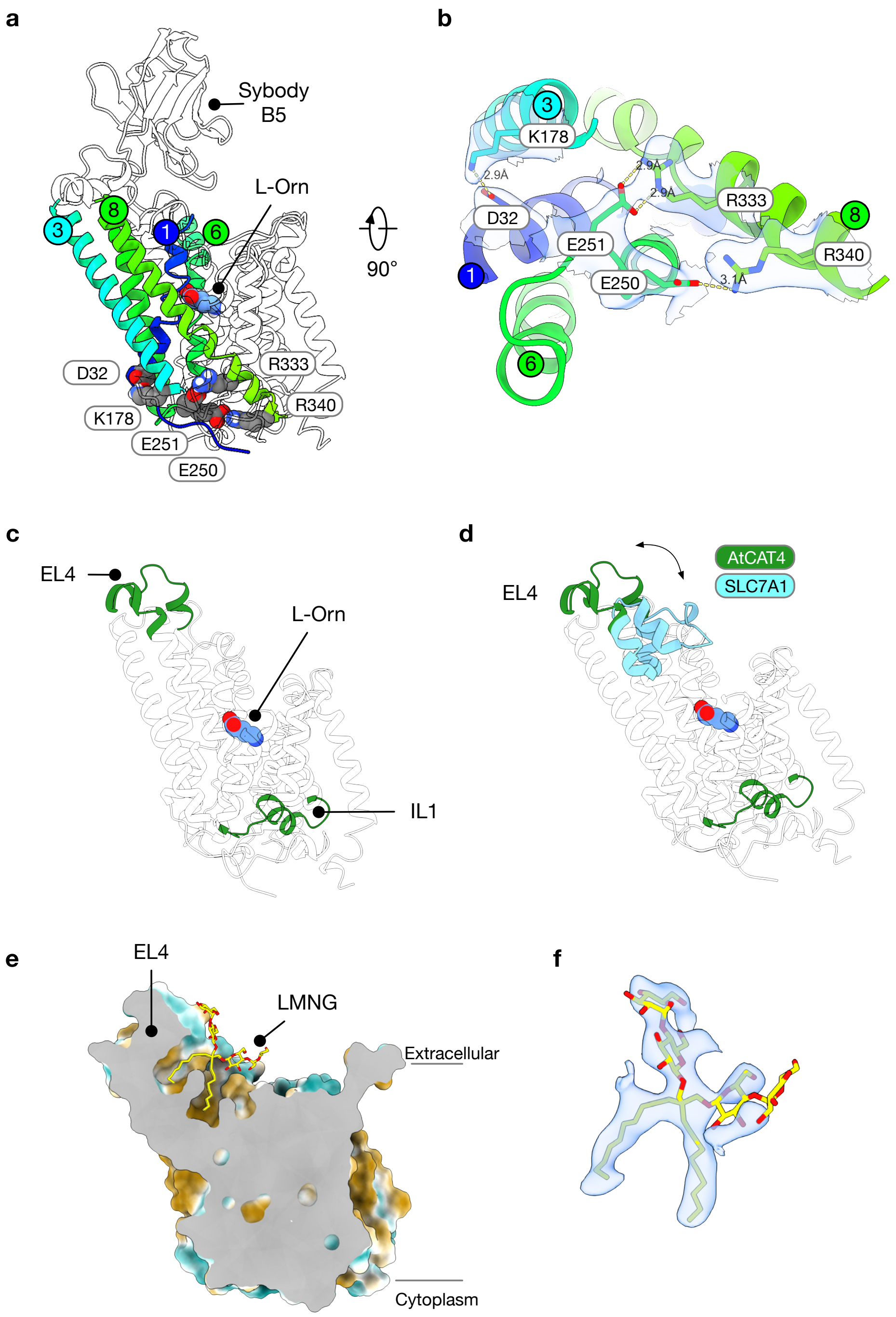


**Supplementary Fig. 4. Structural Analysis of AtCAT4. a.** Cryo-EM structure of AtCAt4 showing the cytoplasmic salt bridge network stabilising the closed state of the transporter. **b.** Zoomed in view of the cytoplasmic salt bridge network showing the cryo-EM density (blue and threshold 0.01). **c.** Structure of AtCAT4 bound to L-Orn showing the location of EL4 and IL1. **d.** Structural comparison of EL4 between AtCAT4 (green) and MmCAT1/SLC7A1 (blue; PDB: 9FQT). The arrow indicates the movement of loop EL4 between the MmCAT1 and AtCAT4 structures. **e.** Hydrophobic surface representation of AtCAT4 showing the location of the bound LMNG detergent molecule. **f.** Zoomed in view of the LMNG molecule shown in e. with the cryo-EM density (blue and threshold 0.02).

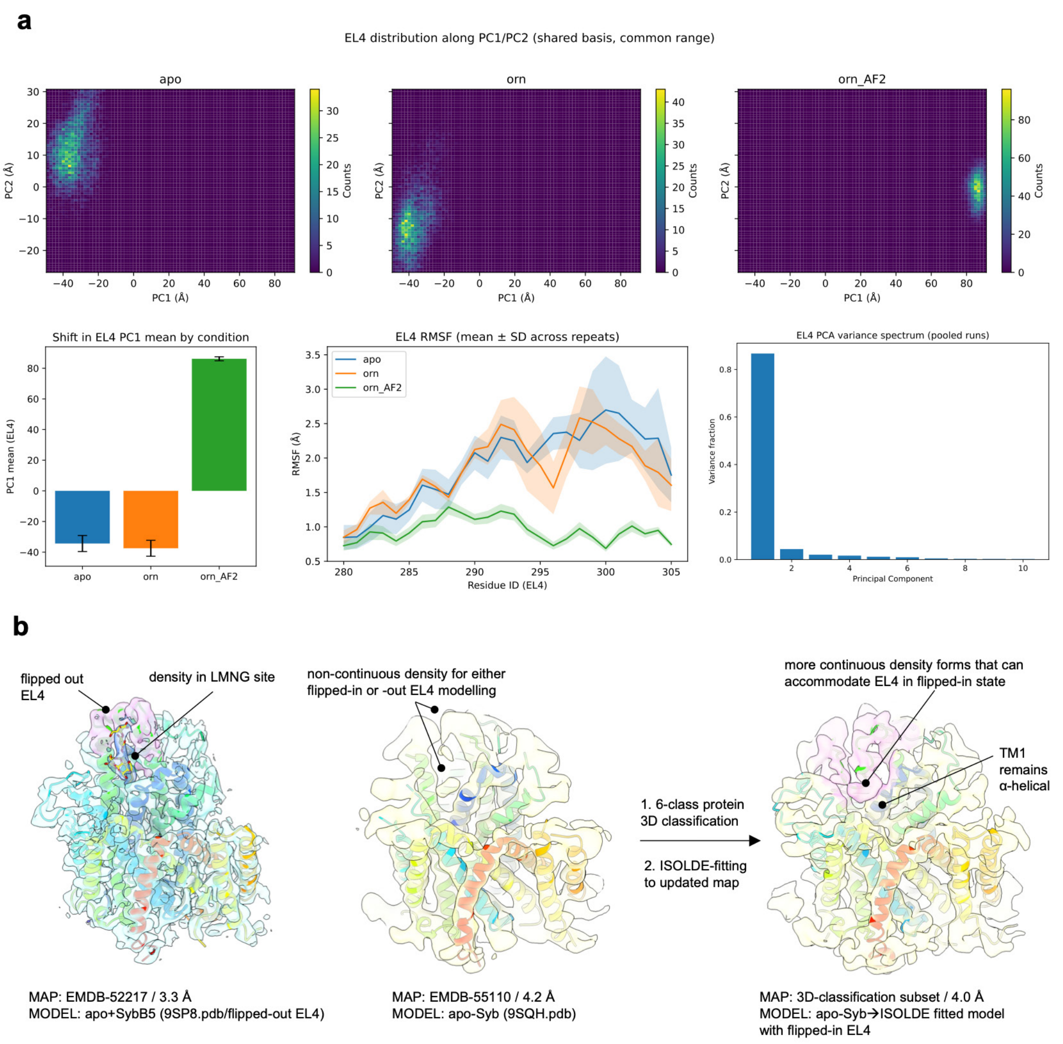


**Supplementary Fig. 5. Molecular Dynamics and cryo-EM analyses support the conformational heterogeneity observed in the EL4 loop. a.** Essential-dynamics/PCA of EL4 backbone coordinates using a shared basis across simulations of the outward-open cryo-EM starting model (apo, $n$ = 3; +L-Orn, $n$ = 3) and an inward-facing AlphaFold2-derived model with EL4 “flipped-in” (+L-Orn, $n$ = 3). 2D projections (PC1/PC2) show that the dominant mode (PC1; >80% variance) corresponds to an EL4 flip-out → flip-in coordinate, separating the outward-open ensembles from the inward-facing ensemble. The PC1 mean is similar for apo and +L-Orn outward-open simulations. Per-residue EL4 Cα RMSF (mean ± SD across repeats) is not detectably changed by ligand in the outward-open state, and is higher than in the inward-facing ensemble, consistent with solvent exposure when EL4 is flipped out and constrained against TM1b when flipped in. The PCA variance spectrum is shown for the pooled trajectories. **b.** Structural context for the EL4 states. Representative outward-open cryo-EM model shows EL4 flipped out and a detergent-sized cavity adjacent to TM1/TM7. The low-resolution −Syb dataset (LMNG) lacks interpretable EL4 density, consistent with mobility. Preliminary EL4-focused 3D classification of the −Syb particle stack yields a minor class (34,329/120,868 particles, 28%) with an alternative, more flipped-in EL4 placement packed against TM1b. Density threshold shown at 0.1.

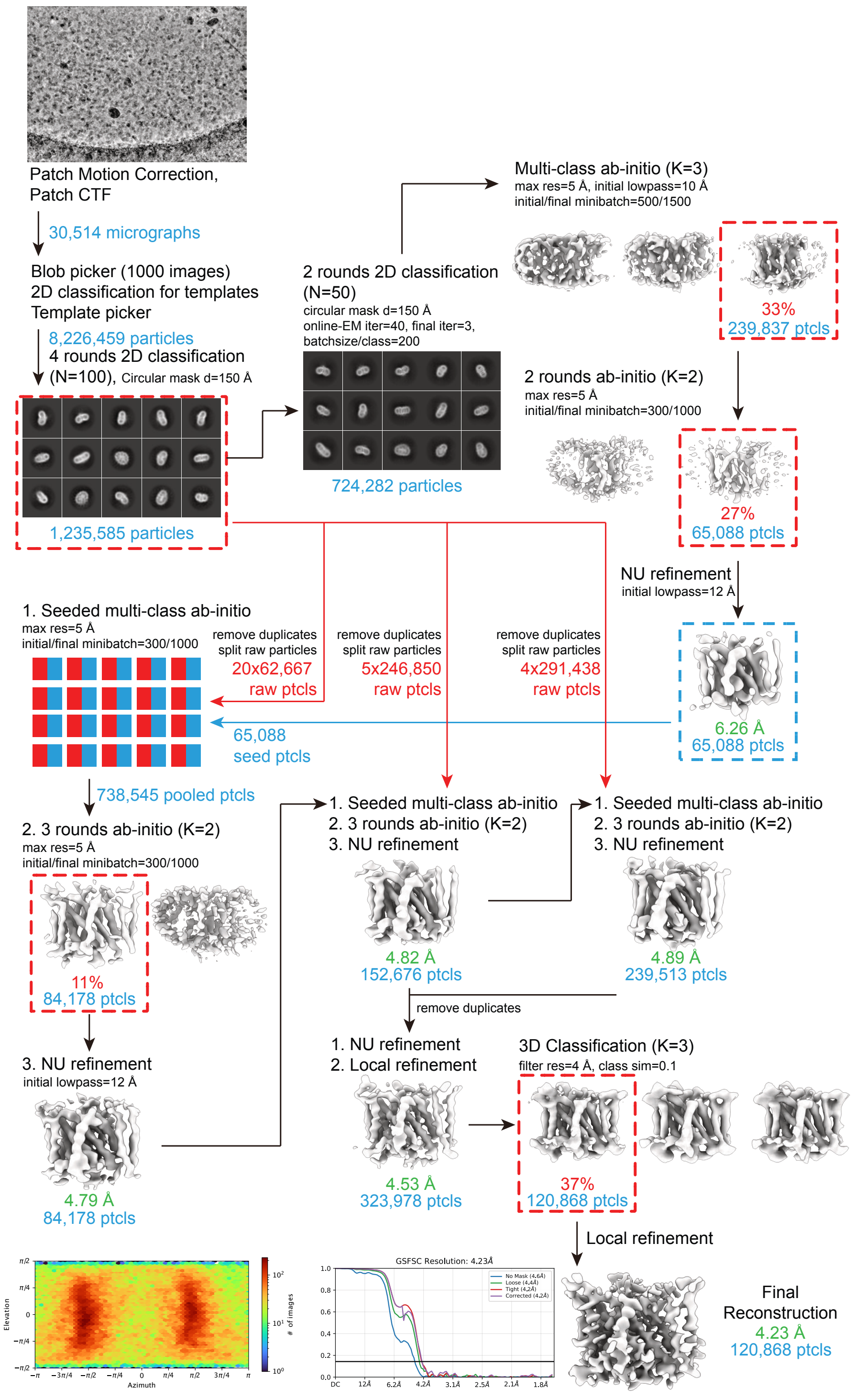


**Supplementary Fig. 6. Cryo-EM processing pipeline for Apo AtCAT4 without sybody B5 (SybB5).** Seeded multi-class ab-initio was performed using particles from NU refinement as "seeds" (blue) into split stacks of "raw" particles (red).

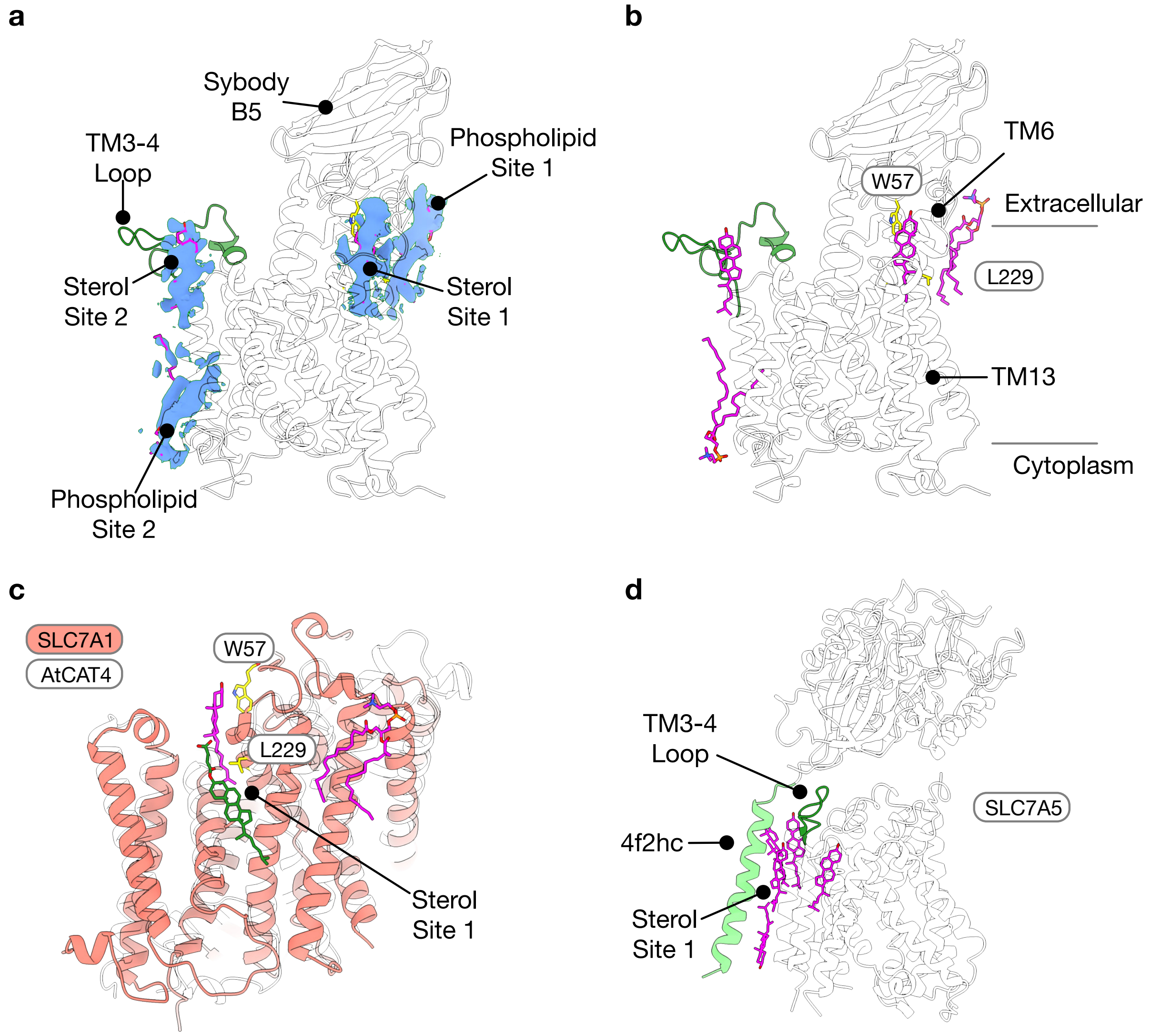


**Supplementary Fig. 7. Analysis of lipid-like densities in AtCAT4 structure. a.** Cryo-EM structure of AtCAT4 with sharpened cryo-EM density for the bound lipid molecules shown (blue and threshold 0.025). **b.** Equivalent view as panel b, but without the cryo-EM density. The main interactions with the sterol in site 1 are labelled. **c.** Overlay of the MmCAT1 (SLC7A1) structure (PDB: 9FQT; pink) onto the AtCAT4 structure (SLC7A4; clear), revealing the conservation of the site 1 sterol binding pocket. **d.** Cryo-EM structure of SLC7A5 (LAT1; PDB: 6JMQ) showing the sterol binding sites in a similar position to site 2 in AtCAT4.

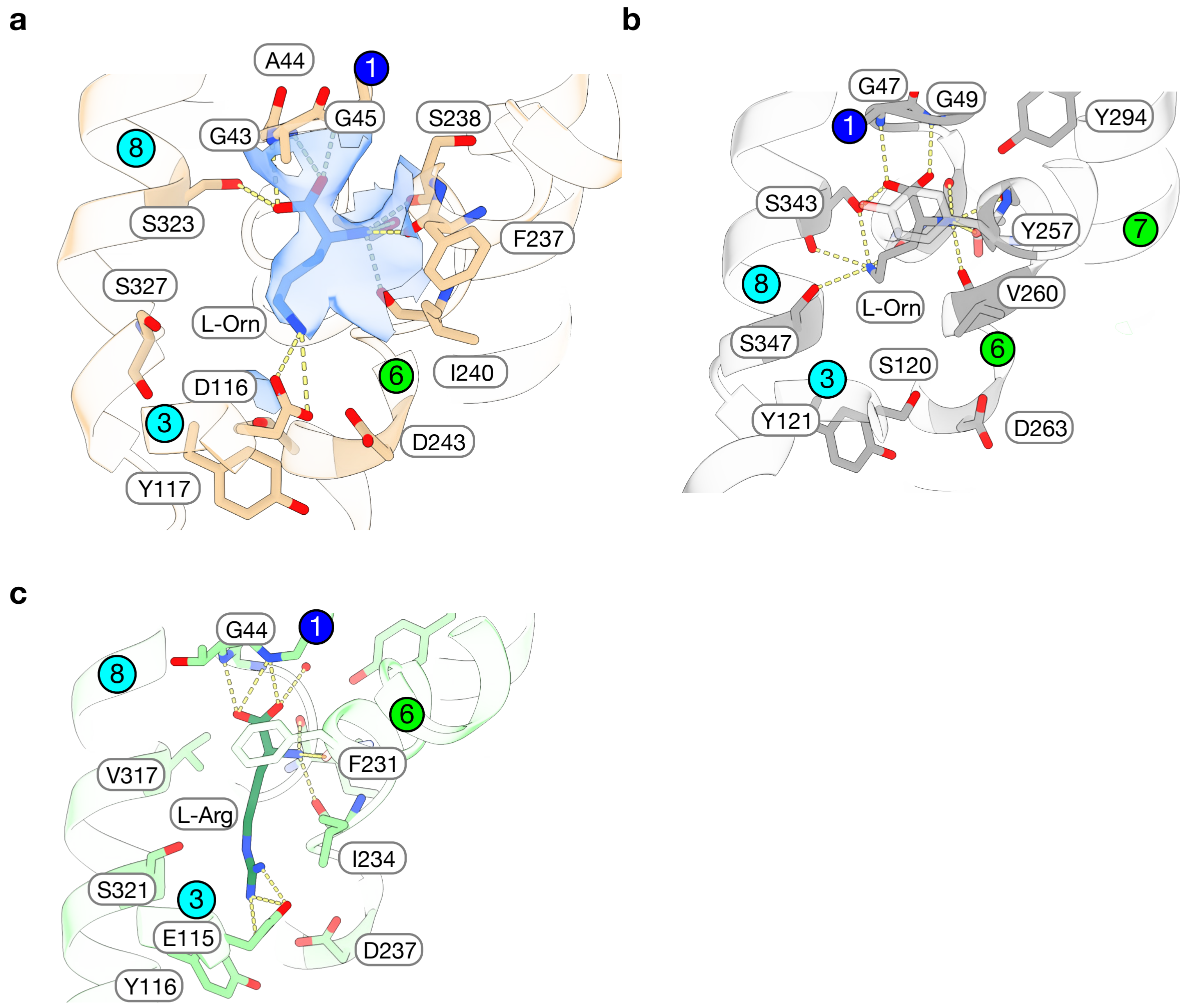


**Supplementary Fig. 8. Structural comparison of amino acid binding in AtCAT4 to GkApcT and MmCAT1 (SLC7A1). a.** The binding site showing the interaction with L-Orn. Key residues interacting with the substrate are shown as sticks, and hydrogen bonds are represented with dashed lines. Cryo-EM density for the L-Orn is shown (blue), contoured at 0.09. Replicated from Fig. 2d for comparison. **b.** Equivalent view of the amino acid binding site in MmCAT1 (SLC7A1) (PDB: 9FQW) bound to L-Ornithine (L-Orn). **c.** Equivalent view of the amino acid binding site in GkApcT (PDB: 6F34) bound to L-Arginine (L-Arg).

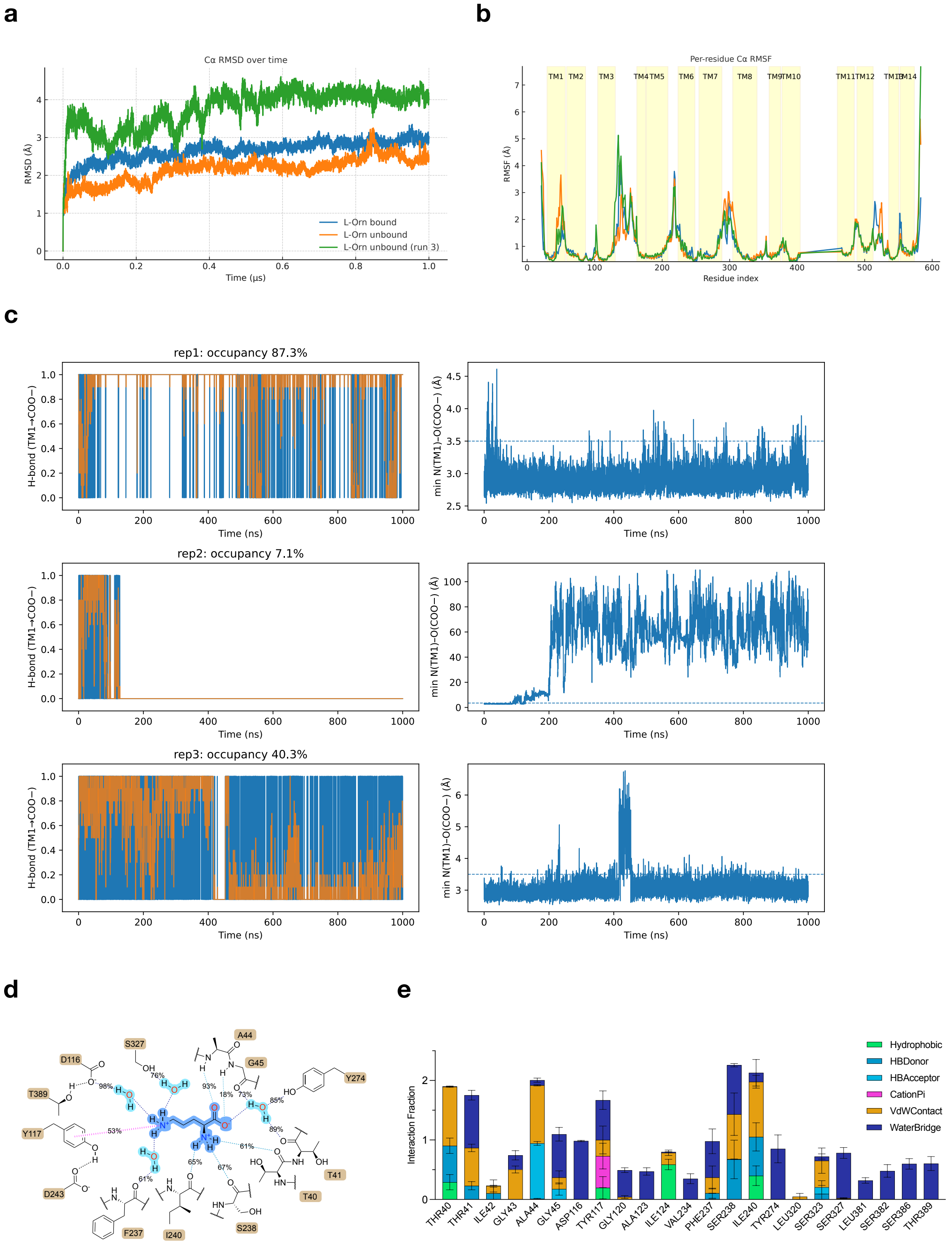


**Supplementary Fig. 9. Molecular Dynamics simulations (3×1 µs and 5×200 ns) of L-Ornithine bound to AtCAT4. a.** Cα root mean square deviation (RMSD) calculated over the whole protein sequence, quantifying AtCAT4 deviation from initial conformation (in Å) over simulation time [excluding IL5 (residues 406-464), which is an unstructured 58-residue domain built as a loop for MD]. Protein least squares were fitted on the stable hash and gate domains (residues 87-224, 282-405, 465-579). **b.**

Per-residue Cα root mean square fluctuation (RMSF) calculated over the whole protein sequence, excluding IL5 and fitting as in **a**. Residues belonging to transmembrane helices 1-14 are shown. **c.** Time-series analysis of the L-Orn carboxylate engagement with TM1 (anchor residue Ala44) for each 1 μs MD replicate (rep1-3). Left panel (per rep): binary trace indicating presence (1) or absence (0) of a direct H-bond where the ligand acts as acceptor (HBAcceptor) from the TM1 backbone N–H (blue line). The 10-window rolling average of the binary trace is shown (orange line). The H-bond occupancy over simulation time is indicated per run. Right panel (per rep): minimum heavy-atom distance between Ala44 backbone N and one of the L-Orn carboxylate O atoms (whichever is closer as they are chemically degenerate); the dashed line at y=3.5 Å is a reference donor–acceptor distance consistent with typical N–H···O hydrogen-bond geometry used throughout in this work's H-bond analyses. **d.** 2d interaction schematic quantifying (in % total simulation time) pairwise interactions between L-Orn and AtCAT4 averaged from 5×200 ns independent repeats (dashed line colours as in **e**). The 5 reps were seeded from the most representative binding site configuration from 1 μs rep 1 (residues within 10 Å of L-Orn, cutoff 1Å with the linkage method). **e.** Bar chart showing the per-residue interaction profile averaged from the simulation replicates. n = three independent 200 ns replicates seeded from the cluster median and from a single 1000 ns simulation; errors shown are s.d.

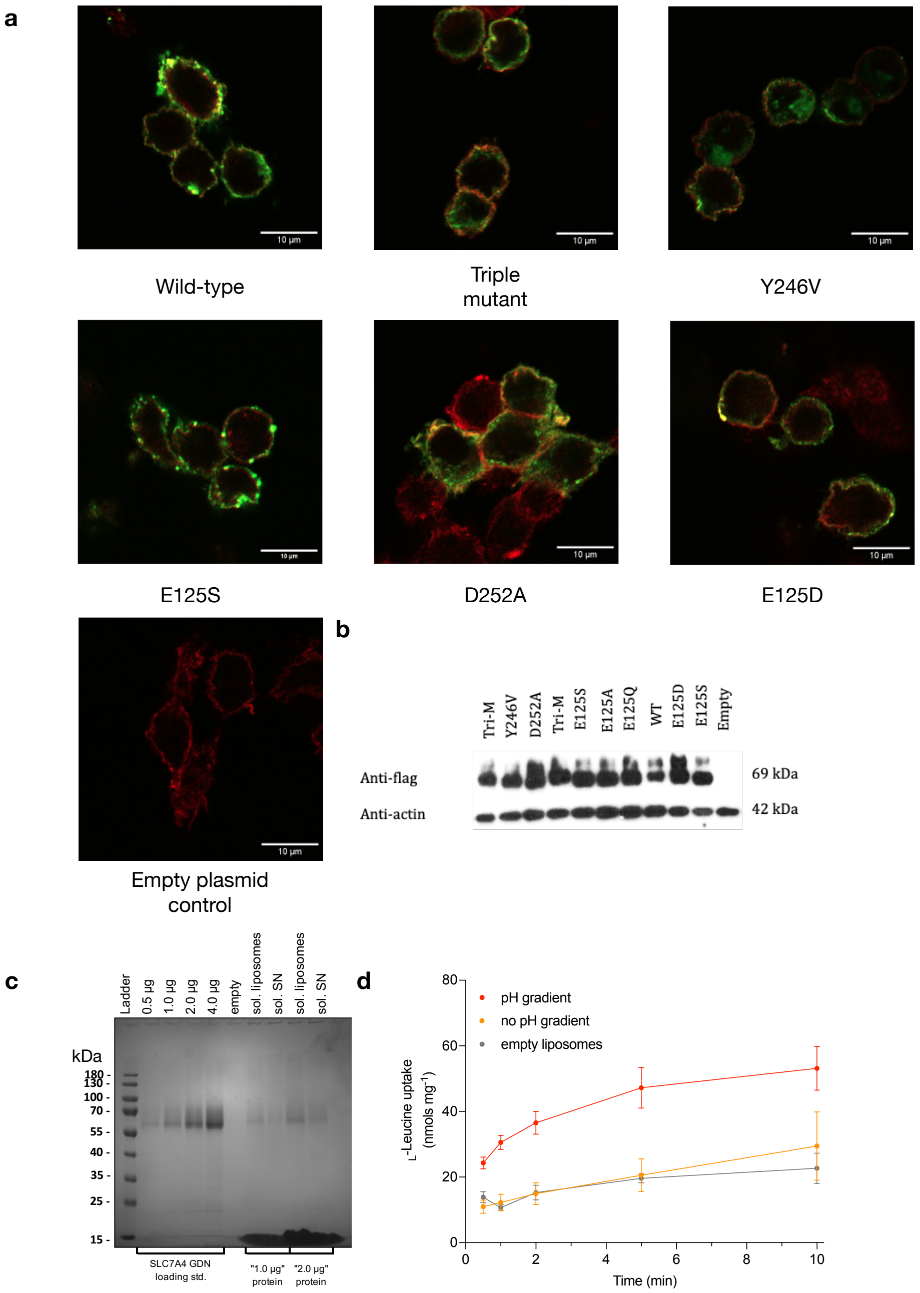


**Supplementary Fig. 10. Functional characterisation of Hs SLC7A4. a.** Immunofluorescence staining of Hs SLC7A4 overexpressed in HCT-116 SLC7A5 KO cells. The green channel shows the localisation of C-terminally FLAG-tagged Hs SLC7A4, imaged with an anti-FLAG antibody; the red channel shows the plasma membrane Na+/K+-ATPase. Images are representative of three independent experiments and taken using a 40x objective. **b.** Western blot analysis of the WT and mutant SLC7A4 used in the cell-based uptake assays using anti-FLAG antibody for Hs SLC7A4 and anti-actin as a loading control. **c.** SDS-PAGE analysis of human SLC7A4 reconstituted into liposomes. **d.** L-Leucine ($^{14}$C L-Leu) uptake into liposomes containing human SLC7A4 either with or without an acetate-induced pH gradient

(outside acidic). n = three independent experiments; errors shown are standard deviations (s.d.).

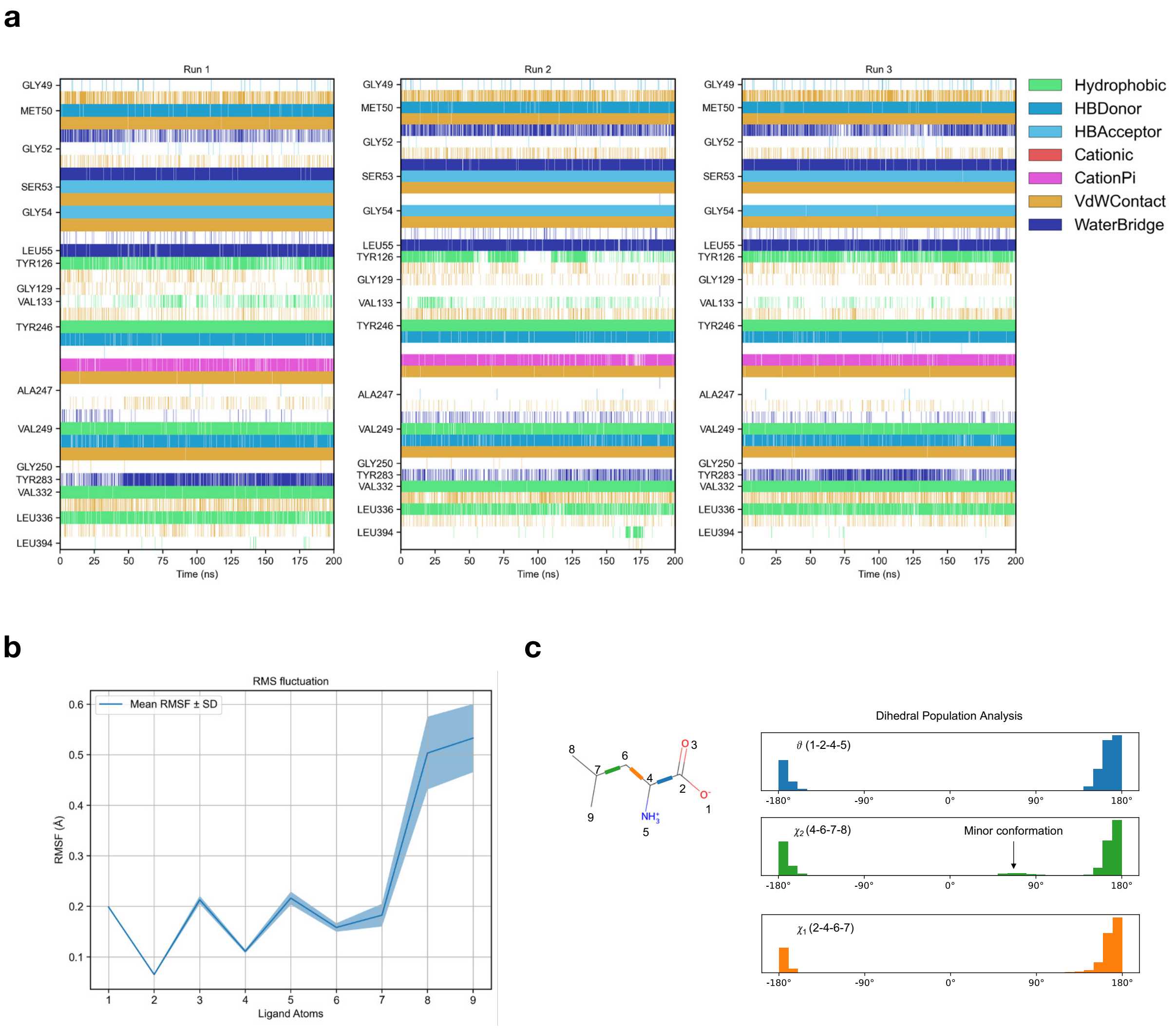


**Supplementary Fig. 11. Molecular dynamics simulations of the L-Leucine bound Human SLC7A4 homology model. a.** Time-resolved per residue interaction analysis; all recorded interactions shown for each independent run. **b.** L-leu RMSF plot showing stable ligand binding over 3 replicates. A modest increase in RMSF is seen for atoms 8 and 9, attributed to a small population of 90° rotation around the dihedral (4-6-7-8; marked in green), shown in panel c. **c.** L-Leu dihedral conformational analysis sampled from the 3 200 ns replicates with $\theta$ referring to the backbone dihedral formed by atoms 1-2-4-5 in the L-Leu structure, $\chi_1$ and $\chi_2$ reporting the dihedrals for the isopropyl sidechain atoms ($\chi_1$=2-4-6-7, $\chi_2$=4,6,7,8). A relatively minor $\chi_2$ ~60° versus the major 180° explains the RMSF>0.5 Å for atoms 8 and 9 in panel b.

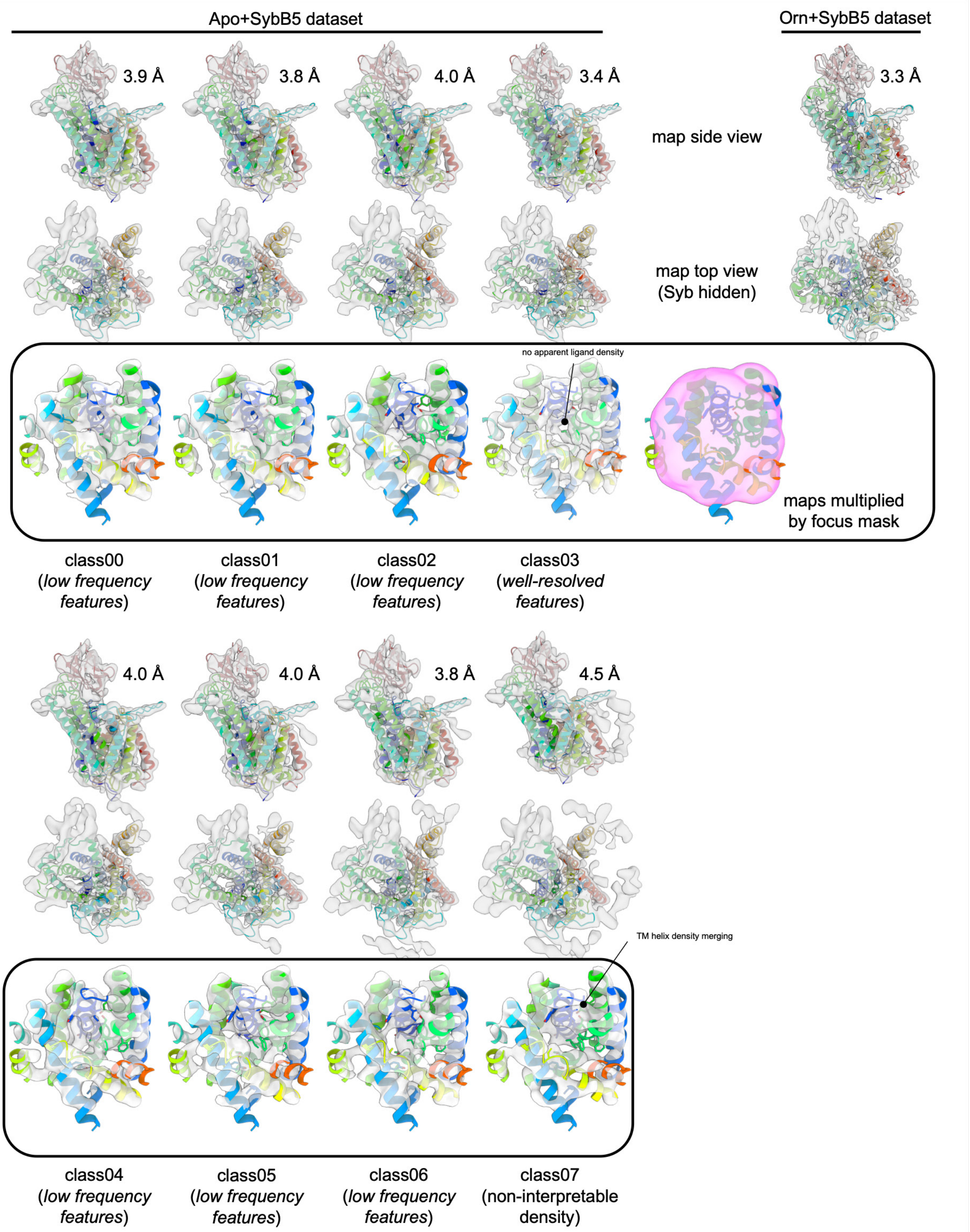


**Supplementary Fig. 12. Focused 3D classification of the apo + SybB5 dataset as a negative control for ligand-density selection.** To control for spurious ligand-like density using our cryo-EM image processing workflow, we applied the same focused 3D classification strategy used for the L-Orn dataset to the apo + SybB5 dataset (no added ligand), using the same aligned focus mask and classification parameters (K = 8; filter resolution = 2 Å; O-EM batch size per class = 10,000; learning rate = 1; initialisation = PCA; class similarity = 0.1; force hard classification = true). For each

class, side and top views are shown; maps displayed in the binding-site panels are multiplied by the focus mask. All class maps were normalised identically, low-pass filtered to 4.0 Å for cross-class comparison (except class07, which was refined to 4.5 Å), and contoured at the same level ($\sigma = 8.5$). Class03 shows the most interpretable feature-rich class, yet does not display ligand-shaped density in the canonical pocket. Classes 0-2 and 4-6 show markedly weaker, low-SNR density in the same region, lacking interpretable sidechain features. We therefore consider these classes unsuitable for atomistic interpretation, and any apparent density in the binding pocket is unreliable for ligand modelling. Class 07 showed the most spurious density, failing to trace the helical backbone of the binding site.

**Supplementary Table 1. Cryo-EM data collection, refinement, and validation statistics**

| | AtCAT4 apo + SybB5 (EMDB-52217) (PDB 9HJK) | AtCAT4 L-Orn bound + SybB5 (EMDB-55065) (PDB 9SP8) | AtCAT4 apo (EMDB- 55110) (PDB 9SQH) |
|---|---|---|---|
| **Data collection and processing** | | | |
| Magnification | 105,000 | 105,000 | 105,000 |
| Voltage (kV) | 300 | 300 | 300 |
| Electron exposure (e–/Å$^2$) | 58.0 | 50.0 | 39.9 |
| Defocus range (μm) | -2.0 to -1.0 | -2.0 to -0.8 | -2.0 to -0.8 |
| Pixel size (Å) | 0.832 | 0.4175 | 0.832 |
| Symmetry imposed | C1 | C1 | C1 |
| Initial particle images (no.) | 1,610,603 | 5,444,589 | 8,226,459 |
| Final particle images (no.) | 642,325 | 44,769 | 120,868 |
| Map resolution (Å)<br>FSC threshold | 3.3<br>0.143 | 3.3<br>0.143 | 4.2<br>0.143 |
| **Refinement** | | | |
| Initial model used (PDB code) | At SLC7A4/CAT4 Alphafold2 entry (Q8W4K3) | 9HJK | 9HJK |
| Model composition in the asymmetric unit<br>Non-hydrogen atoms<br>Protein residues<br>Ligands | <br><br>4818<br>631<br>98 | <br><br>4965<br>633<br>240 | <br><br>3193<br>433<br>0 |
| Average *B* factors (Å$^2$)<br>Protein<br>Ligand<br>Waters | <br>90.83<br>—<br>58.98 | <br>98.74<br>111.87<br>70.15 | <br>77.76<br>—<br>— |
| R.m.s. deviations<br>Bond lengths (Å)<br>Bond angles (°) | <br>0.005<br>0.793 | <br>0.004<br>0.615 | <br>0.002<br>0.583 |
| Validation<br>MolProbity score<br>Clashscore<br>Poor rotamers (%) | <br>1.58<br>7.68<br>0.81 | <br>1.46<br>5.33<br>0.61 | <br>1.56<br>6.86<br>0.00 |

**Supplementary Table 2. Molecular dynamics data table**

| | System | Starting Model | Replicates × Time | Referenced | Deposited Files |
|---|---|---|---|---|---|
| **1** | AtCAT4 (Apo) | 9HJK | 3 × 1000 ns | rep 1-3 referenced as “Apo” in Fig 2<br>rep 3 data used in Fig 2b | • Apo_1000ns/initial_config.gro<br>• Apo_1000ns/rep1_whole_nojump_center_rotxyz-transxyz.pdb<br>• Apo_1000ns/rep2_whole_nojump_center_rotxyz-transxyz.pdb<br>• Apo_1000ns/rep3_whole_nojump_center_rotxyz-transxyz.pdb<br>• Associated .tpr, .topol and .mdp files |
| **2** | AtCAT4 (L-Orn) | 9SP8 | 3 × 1000 ns | rep 1: L-Orn remains bound - data used in Fig 2d, ED Fig 9a-c<br>rep 2: L-Orn diffuses from the binding site - data used in ED Fig 9a-c<br>rep 3: site L-Orn disengages TM1 - data used in Fig 2c, ED Fig 9a-c | • Orn_1000ns/initial_config.gro<br>• Orn_1000ns/rep1_whole_nojump_center_rotxyz-transxyz.pdb<br>• Orn_1000ns/rep2_whole_nojump_center_rotxyz-transxyz.pdb<br>• Orn_1000ns/rep3_whole_nojump_center_rotxyz-transxyz.pdb<br>• Associated .tpr, .topol and .mdp files<br>• Zwitterionic L-ornithine (ORN) GAFF2/RESP parameterisation files |
| **3** | AtCAT4 (L-Orn) | Seeded from the median binding site configuration of system **2** | 5 × 200 ns | rep 1-5 data pooled and used in ED Fig 9d-e | • Orn_200ns/initial_config.gro<br>• Orn_200ns/rep1_whole_nojump_center_rotxyz-transxyz.pdb<br>• Orn_200ns/rep2_whole_nojump_center_rotxyz-transxyz.pdb<br>• Orn_200ns/rep3_whole_nojump_center_rotxyz-transxyz.pdb<br>• Orn_200ns/rep4_whole_nojump_center_rotxyz-transxyz.pdb<br>• Orn_200ns/rep5_whole_nojump_center_rotxyz-transxyz.pdb |

| | | | | | |
|---|---|---|---|---|---|
| | | | | | • Associated .tpr, .topol and .mdp files<br>• Zwitterionic L-ornithine (ORN) GAFF2/RESP parameterisation files |
| **4** | Hs SLC7A4 (L-Leu) | Homology model (HM) of 9SP8 | 1 × 1000 ns | HM and system equilibration; unrestrained – used to equilibrate L-leu-bound HM to seed system **5** | • HM_1000ns/initial_config.gro<br>• HM_1000ns/pr1000ns_whole_nojump_center_rotxyz-transxyz.pdb<br>• Associated .tpr and .mdp files<br>• Zwitterionic L-leucine (ZL) parm99sb "Horn" parameterisation files (AMBER and Gromacs formats) |
| **5** | Hs SLC7A4 (L-Leu) | Seeded from the median binding site configuration of system **4** | 3 × 200 ns | rep 1-3 data pooled and used in ED Fig 11 a-c | • HM_200ns/initial_config.gro<br>• HM_200ns/rep1_whole_nojump_center_rotxyz-transxyz.pdb<br>• HM_200ns/rep2_whole_nojump_center_rotxyz-transxyz.pdb<br>• HM_200ns/rep3_whole_nojump_center_rotxyz-transxyz.pdb<br>• Associated .tpr, .topol and .mdp files<br>• Zwitterionic L-leucine (ZL) parm99sb "Horn" parameterisation files (AMBER and Gromacs formats) |
| | | | **8.6 μs total simulation time** | | All forcefield, topology and parameter files are deposited in Zenodo [DOI: 10.5281/zenodo.17184930]. |
